# Evaluation of functionality and quality of life in patients with lower limb fractures after traffic accidents admitted to IOT-HCFMUSP

**DOI:** 10.1016/j.clinsp.2025.100742

**Published:** 2025-08-05

**Authors:** Aline Ferreira Guimarães Gubolin, Julia Maria D'Andrea Greve, Vanderlei Carneiro da Silva

**Affiliations:** a Instituto de Ortopedia e Traumatologia do Hospital das Clínicas da Faculdade de Medicina da Universidade de São Paulo (IOT-HCFMUSP), São Paulo, SP, Brazil; b Department of Orthopedics and Traumatology, Faculdade de Medicina da Universidade de São Paulo (FMUSP), São Paulo, SP, Brazil; c Movement Studies Laboratory (LEM), Faculdade de Medicina da Universidade de São Paulo (FMUSP), São Paulo, SP, Brazil; d Postgraduate Programs in Experimental Pathophysiology and Musculoskeletal System Sciences at Faculdade de Medicina da Universidade de São Paulo (FMUSP), São Paulo, SP, Brazil; e Movement Studies Laboratory (LEM), Instituto de Ortopedia e Traumatologia do Hospital das Clínicas da Faculdade de Medicina da Universidade de São Paulo (IOT-HCFMUSP), São Paulo, SP, Brazil

**Keywords:** Traffic accident fractures quality of life return to work

## Abstract

**Introduction:**

Traffic accidents pose a significant public health concern, particularly for motorcyclists, who are especially susceptible to severe and debilitating injuries. These injuries can impair work activities and diminish the overall quality of life.

**Objective:**

This study aims to assess the functionality, quality of life, and return-to-work status of patients who sustained lower limb fractures due to traffic accidents.

**Methods:**

The authors conducted a retrospective observational study involving patients hospitalized with lower limb fractures due to traffic accidents at the Institute of Orthopedics and Traumatology at the Hospital das Clínicas, Faculty of Medicine, University of São Paulo, from 2018 to 2022. The authors performed the Timed Up and Go (TUG) test, and the 10-meter walk test, and administered the SF-12 questionnaire to evaluate quality of life. Additional questions regarding their return to work were also included.

**Results:**

A total of thirty-nine patients were evaluated six months or more following their last surgery. The results indicated that the average TUG time was 12.3 ± 3.3 seconds, and the average speed in the 10-meter walk test was 1.6 ± 0.3 m/s. The SF-12 results revealed a mean score of 48.6 ± 10.9 points in the mental domain and 39.9 ± 9.5 points in the physical domain. Notably, 72 % of patients had not returned to work. The length of hospital stay and the severity of injuries were significant factors influencing patient recovery.

**Conclusion:**

Although patients demonstrated normal functional status, the majority reported difficulties in performing daily activities and experienced low quality of life even after rehabilitation. This highlights the considerable impact that traffic accidents have on individuals' lives.

## Highlights

• Complex fractures of the lower limbs, often resulting from traffic accidents, require extended periods of hospitalization and rehabilitation. These injuries are highly disabling and significantly impact the victims' quality of life and work conditions.
• Many individuals experience substantial physical limitations and a decreased quality of life. This suggests that, even after months of rehabilitation, many have not been able to return to their functional level before the accident.
• The severity of the injuries influences the ability to return to work. Factors such as exposed fractures, multiple fractures, and long hospital stays are associated with poorer functional outcomes.

## Introduction

Traffic accidents are one of the main causes of hospitalizations in orthopedic units, require multiple surgical procedures, and cause disabling functional losses^1^

Lower Limb Injuries (LL), one of the most frequent accidents, can require long periods of hospitalization and rehabilitation, with repercussions on the quality of life and working conditions of individuals^2^^,^^3^

Understanding the conditions of victims: severity of injury, access to rehabilitation, and social and labor reintegration are crucial for developing policies to prevent and reduce harm. There is a lack of epidemiological data on the disabilities, quality of life, and labor conditions of patients, justifying the current research, which aimed to evaluate functionality, quality of life, and return to work in patients who suffered lower limb fractures after a traffic accident.

## Methods

This was a retrospective cross-sectional study (CAAE control number: 54259421.0.0000.0068/SGP 22114) and is by the STROBE criteria for cross-sectional studies.

The sample consisted of patients admitted to IOT-HCFMUSP between 2018 and 2022, victims of traffic accidents with lower limb fractures. The inclusion criteria were: individuals of both sexes, aged 18 to 59 years at the time of the accident, traffic accidents with surgical fractures of the lower limbs, six or more months after the last surgery, preserved cognitive function, and signing the Free and Informed Consent Form (FICF). Before the call, medical records were consulted to assess inclusion criteria and clinical data collection. After the initial analysis, patients were invited to participate (by telephone), and appointments were made for those who agreed to participate. In the face-to-face evaluation, the Timed Up and Go (TUG) test, 10-meter Walk Test, and the SF-12 (12-item Short-Form Health Survey) were performed to assess quality of life.

### Data analysis

Categorical variables were described according to frequency distribution in absolute and percentage values; the normality of variable distribution was assessed by the Shapiro-Wilk test (*p* < 0.05). The association of categorical variables was performed using the Chi-Square and Fisher's Exact Tests. Comparisons between return to work and time of external fixator use were performed using Student's *t*-test or Mann-Whitney.

Decision Tree models were constructed to predict the length of hospital stay and return to work. The aim of the analyses was to identify a set of variables associated with return to work. vs. participants who did not return and compare predictors according to length of hospital stay. The data used as predictors included demographic characteristics, quality of life and functionality, accident data, and treatment data. The analyses were conducted in *R* software, version 4.2.2, using the packages “rpart”, “rpart.plot”, “e1071” and “caret”.

Three Decision Tree models were constructed for each outcome, which allowed us to verify the consistency of the predictors when they were included and/or removed and the performance of each model based on the data set that was used, which are described as follows

- Model 1 (length of hospital stay) included the following predictors: Age, gender, ISS, number of surgeries, length of physiotherapy (months), walking speed (m/s), comorbidities, trauma mechanism, whether the driver was involved, occupation, work accident, existence of joint fractures, associated fractures, bilateral involvement, exposed fracture, immediate complications, late complications, new surgery, receipt of INSS benefit;

- Model 2 (length of hospital stay) included the predictors: Age, gender, ISS, number of surgeries, number of steps in the 10 m Walk test, time of physiotherapy (months), characteristics of fractures, whether articular, extra-articular, exposed, closed, multiple, single fractures, and pelvic involvement;

- Model 3 (length of hospital stay) included the predictors: Age, gender, ISS, SF-12 PCS, SF-12 MCS, number of steps in the 10 m Walk test, gait speed (m/s), TUG, time of physiotherapy (months), comorbidities, trauma mechanism, whether driver, occupation, work accident, fracture characteristics, whether joint fractures, associated, bilateral involvement, exposed fracture, immediate complications, late complications, new surgery, INSS benefit, number of surgeries;

- Model 4 (return to work) included predictors: Age, gender, ISS, SF-12 PCS, SF-12 MCS, number of steps in the 10 m Walk test, gait speed (m/s), TUG, time of physiotherapy (months), comorbidities, trauma mechanism, whether driver, occupation, work accident, whether joint fractures, associated, bilateral involvement, exposed, immediate complications, late complications, new surgery, INSS benefit, number of surgeries;

- Model 5 (return to work) included predictors: Age, gender, ISS, number of surgeries, number of steps in the 10 m Walk test, walking speed (m/s), time of physiotherapy (months), characteristics of fractures, whether articular, extra-articular, exposed, closed, multiple, single, pelvic involvement;

- Model 6 (return to work) included predictors: Age, gender, ISS, SF-12 PCS, SF-12 MCS, number of steps in the 10 m TC, gait speed (m/s), TUG, number of surgeries, time of physiotherapy (months).

Based on the set of defined predictors, the algorithm identified the data in a scale of importance for each outcome (length of hospital stay and return to work). At each level of the tree, the model identified one or more predictors, from the set of variables included those that best allow for the division of the sample. The predictors that identify the possibility of a class occurring, from among the possible classes (return to work or not) or the prediction of quantitative values (length of hospital stay), help subdivide the sample through decision nodes. Each node can have two or more branches, and this leads to a leaf of the tree. For a decision to occur, the flow begins at the root, which is the starting point, and the attributes that divide the participants, represented by the nodes of the decision tree, determine the next step of the flow.

In the study, the parameters used to build the models were: split = "information" [division by information gain], minsplit = 5 [minimum number of observations in a node], method = "cv" [cross-validation], maxdepth = 5 [depth level], cp = 0.01 [divisions that do not improve performance should be removed]. The original dataset was subdivided into training and testing, in the proportion of 70 % and 30 %, respectively, using the “sample” function. The “OverSample” function was used to balance the return-to-work classes according to the original database (did not return (*n* = 28, 72 %) vs. returned (*n* = 11, 28 %).

## Results

During the proposed study period, 734 patients who were victims of traffic accidents were hospitalized, 233 of whom had lower limb fractures. Of these, 194 were excluded and the study was then carried out with 39 patients who met the specifications. Table 1 shows the characteristics of the patients.

**Table 1:** Characteristics of patients with fractures and lower limbs admitted to IOT-HCFMUSP between 2018‒2022 who were victims of traffic accidents.

	% (n)	Average	DP
**Gender**			
Masculine	82 % (32)		
Feminine	18 % (7)		
**Age**		33	±8.4
**Trauma Mechanism**			
Bicycle	8 % (3)		
Automobile	10 % (4)		
Run over	13 % (5)		
Motorcycle	69 % (27)		
**Occupation**			
Motorcycle courier	10 % (4)		
Motoboy App	10 % (4)		
Others	72 % (28)		
**Work Accident**			
Yes	51 % (20)		
No	49 % (19)		
**Total length of stay (days)**		13	11
**Physiotherapy time (months)**		5	2
**PO Time (months)**		15	13
**ISS**		3.28	6.41
**Joint fractures**			
Yes	41 % (16)		
No	59 % (23)		
**Associated fractures**			
Yes	36 % (14)		
No	64 % (25)		
**Exposed fracture**			
Yes	51 % (20)		
No	49 % (19)		
**External fixator**			
Yes	49 % (19)		
No	51 % (20)		
**Return to work**			
Yes	28 % (11)		
No	72 % (28)		
**INSS benefit**			
Yes	54 % (21)		
No	46 % (18)		
**Inability to perform activity**			
Yes	77 % (30)		
No	23 % (9)		

%, Percentage; SD, Standard Deviation; ISS, Injury Severity Score; INSS, National Institute of Social Security; PO, Post-Operative.

**Fig. 1:**
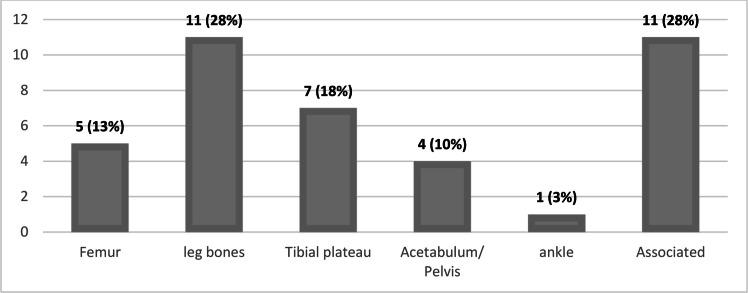
Location of fractures in the lower limbs.

**Fig. 2:**
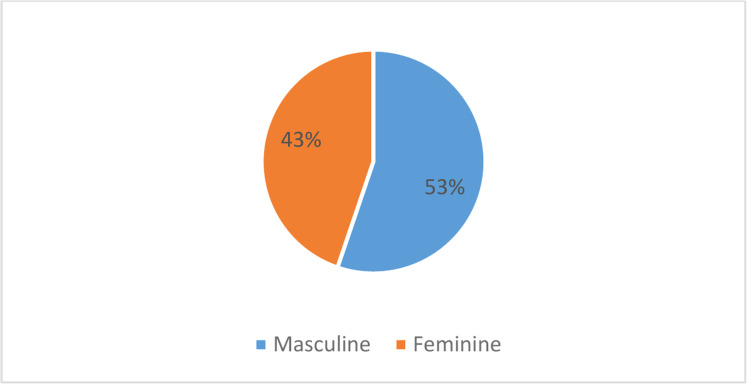
Graph showing the proportion of exposed lower limb fractures according to gender in the study population.

The location of fractures in the lower limbs is seen in Fig. 1 and the proportion of exposed fractures according to gender in Fig. 2.

**Fig. 3:**
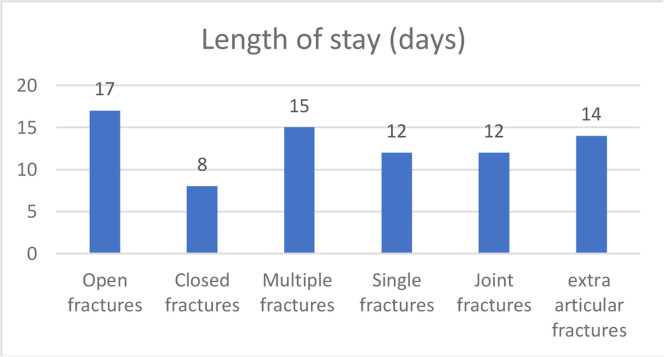
Length of hospital stay for fractures in the studied population.

The length of hospital stay was 13±11 (04‒52) days. Fig. 3 shows the length of hospital stay according to the type of fracture.

The functional data evaluated are shown in Table 2.

**Table 2:** Functional assessment (TUG, gait speed, SF-12 PCS and SF-12 MCS) of the studied sample.

	TUG	SF-12 MCS	SF-12 PCS	Walking Speed (m/s)
**Average**	12.28	48.58	39.92	1.6
**DP**	3.32	10.91	9.50	0.3

SF-12 PCS, SF-12 Physical Component Scores; SF-12 MCS, SF-12 Mental Component Scores; TUG, Timed Up and Go; m/s, Meters per second; SD, Standard Deviation.

### Decision trees

#### Length of hospital stay

**Fig. 4:**
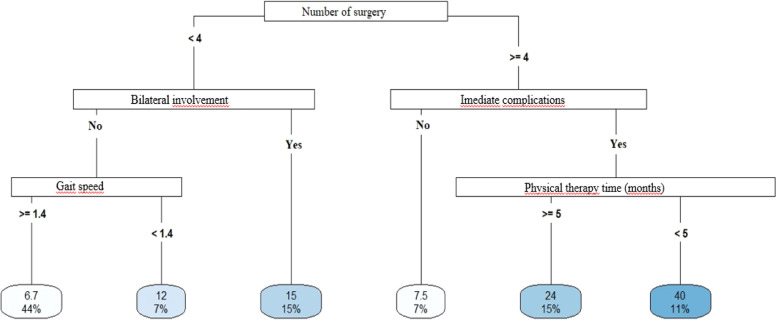
Decision tree for length of stay – model 1. <, less than; ≥, greater than or equal to; ms walking speed, walking speed in meters/seconds.

**Fig. 5:**
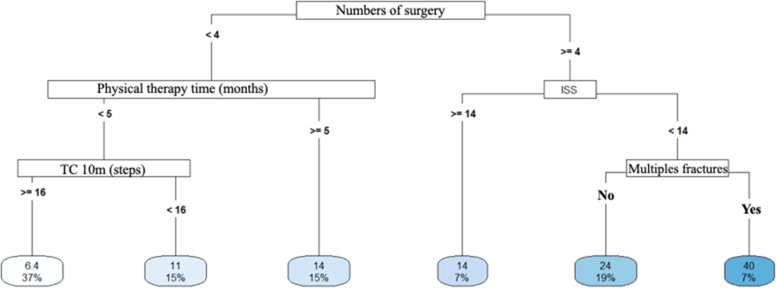
Decision tree for length of stay – model 2. <, less than; ≥, greater than or equal to; TC 10m_steps, number of steps in the 10 m walk test; ISS, injury severity score.

**Fig. 6:**
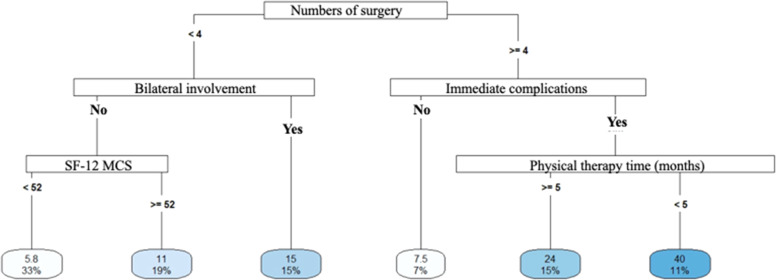
Decision tree for length of stay – model 3. <, less than; ≥, greater than or equal; SF_12_MCS, SF-12 physical domain.

Longer hospital stays were associated with a greater number of surgeries (Fig. 4), multiple fractures (Fig. 5), bilateral injuries and a higher Mental Component (SF-12 MCS) (Fig. 6).

#### Return to work

**Fig. 7:**
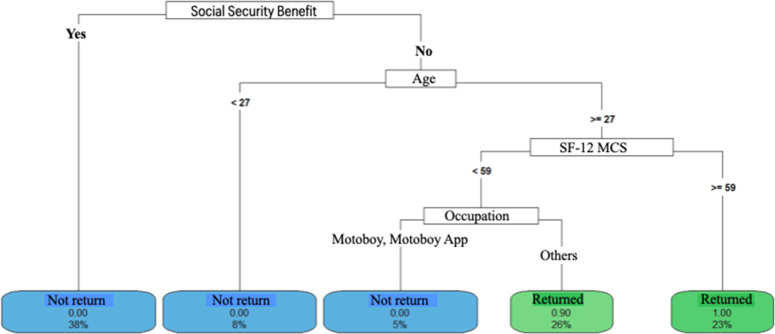
Return to work decision tree – model 4. <, less than; ≥, greater than or equal to; SF_12_MCS, SF-12 mental domain.

**Fig. 8:**
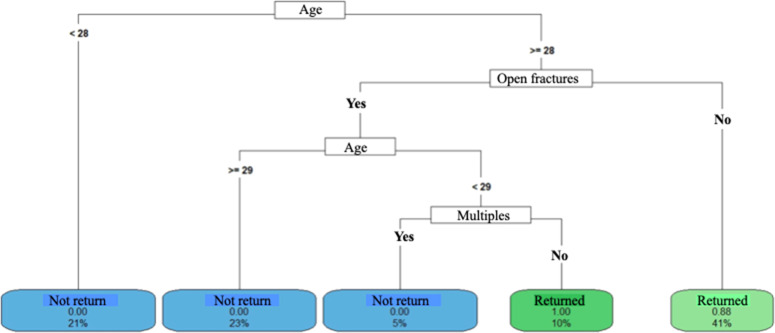
Return to work decision tree model 5. <, less than; ≥, greater than or equal to.

**Fig. 9:**
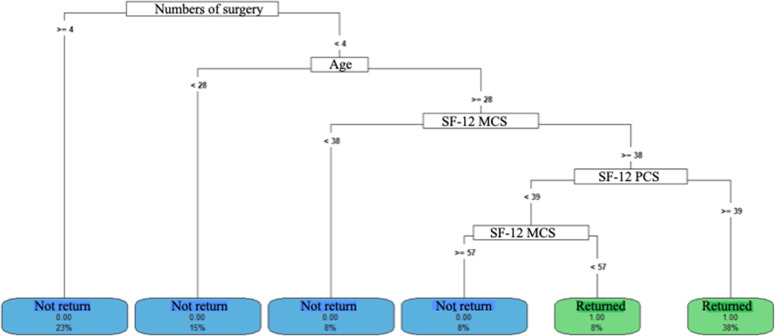
Return to work decision tree model 6. <, lower; ≥, greater than or equal; SF-12 MCS, SF-12 mental domain; SF-12 PCS, SF-12 physical domain.

Patients with social security did not return to work (Figs. 7, 8 and 9). Those without social security had varied behaviors: individuals under 27-years-old, non-motorcycle couriers, and SF-12 MCS greater than 59-points returned to work more often (Fig. 7). Exposed fractures and a greater number of surgeries were related to a lower return to work (Figs. 8 and 9).

## Discussion

The results show a predominance of young men (82 %) and motorcycle accidents (69 %). It repeats the pattern of previous studies: motorcyclists with serious injuries, due to greater exposure to risk, with a predominance of leg bone fractures^1^^,^^4^, ^5^, ^6^, ^7^, ^8^

In this study, the exposed and multiple fractures had a longer hospital stay of 17±8 days, data in agreement with the literature: the greater the severity, the longer the hospital stay and rehabilitation. Canônica et al.^4^, reported that patients with more severe injuries had longer treatment times and greater disability. Patients with unilateral fractures and fewer surgeries spent less time hospitalized and had better functional performance (speed of normal gait).

### Functional tests

#### TUG

The TUG was within the standards considered normal (12.3 ± 3.3 seconds)^9^, but possibly this was not a sensitive test to detect limitations associated with fractures, being not very suitable for the young population, and also due to the different types and different locations of the fractures in the current study.

#### 10m walk test

The speed of walking also showed normal values, but possibly due to the effect of unilateral and less severe fractures. The four or 10-meter walk test, in patients after surgery and lower limb fractures, is considered reliable and reproducible and can be used for clinical and functional evaluation, as already demonstrated in a previous study^10^

### Quality of life

The SF-12 questionnaire showed low physical (PCS 39.9 ± 9.5) and mental (MCS 48.6 ± 10.9) domain values, denoting the compromised quality of life and the impact that accidents cause to individuals, highlighting the great compromise of the mental domain.

All the individuals were evaluated with at least six months after the last surgical intervention, and it was observed that the deleterious effects on quality of life persist. Kaske et al.^11^, in 2014, used the SF-36 in patients with lower limb fractures and showed a decrease in all domains, especially in relation to pain and activities of daily living, a result in agreement with those of the current study.

One notable factor is that, even if individuals have a functional assessment of normal gait and balance, the impact on the performance of daily life activities and loss of quality of life persist, showing the serious consequences of traffic accidents.

The longer hospital stay is related to the severity of the injuries: exposed, multiple, bilateral fractures, and with a greater number of surgeries, which would be expected.

### Return to work

In this study, 72 % of patients had not returned to work.

The social security factor was an important predictor of not returning to work (38 %), regardless of other factors. Among those who did not receive social security, over 27-years of age, 43 % returned to work, but some factors should be highlighted: better mental condition (23 %) and not being a motorcycle courier (20 %). Better mental conditions and activities with less physical demand justify the greater return to work, although the lack of social security coverage is an important predictor.

In a systematic review of the socioeconomic impact of orthopedic trauma, O'Hara et al.^12^, in 2020, pointed out that one-third of the patients in the included studies did not return to work activities within one year after the trauma, corroborating the present study, where 72 % of the individuals did not return to work and, these individuals had an average postoperative time of 15-months. As factors that influence the return to work, this cited study points out the severity of the injury, patient comorbidities, and type of pre-injury employment, also agreeing with the present study, where the severity of the injury was a determining factor in the results.

Studies indicate that rehabilitation interventions include physical and psychological approaches and person-centered activities, as well as personalized goals, so that a better functional result can be obtained, thus facilitating the return to work and daily activities and contributing to the effectiveness of interventions^13^, ^14^, ^15^

### Study limitations

The limitations of the study are the sample size (many refusals to participate), the retrospective nature of the study, and the bias given by the difference in evaluation time in relation to trauma and surgery.

However, the results show that lower limb fractures cause serious disabilities and point to the need for preventive interventions and more effective rehabilitation programs for traffic accident victims, which involve monitoring by a specialized multidisciplinary team to deal with the demands of this patient profile, aiming to reduce their long-term consequences on the functionality and quality of life of those affected.

## Conclusions

Patients with lower limb fractures after traffic accidents who remained hospitalized show a loss of quality of life in the physical and mental domains, even after discharge from treatment.

Despite normal functional tests, many faces significant limitations in the physical domain of quality of life, indicating that even after months of rehabilitation, they have not yet managed to return to their pre-accident functional level.

Most patients in this study did not return to work, which shows the impact of injuries on daily life and how injury severity, age, and occupation can be factors that influence this non-return and play crucial roles in functionality, work and return to active life. Specific rehabilitation programs for each type of fracture, focused on individual needs and also including psychological and social support, may be more effective in recovering functional capacity and thus providing a higher rate of return to work.

The findings of this study are representative of the population studied, but require more similar and more robust studies to validate them.

## CRediT authorship contribution statement

**Aline Ferreira Guimarães Gubolin:** Conceptualization, Methodology, Validation, Investigation, Writing – original draft. **Julia Maria D'Andrea Greve:** Conceptualization, Methodology, Validation, Data curation, Writing – review & editing. **Vanderlei Carneiro da Silva:** Software, Validation, Formal analysis.

## Declaration of competing interest

There were no conflicts of interest in the study, and there was also no funding for the research. The assessments are already routinely performed in rehabilitation, so there were no costs for the researchers or the Institution.
